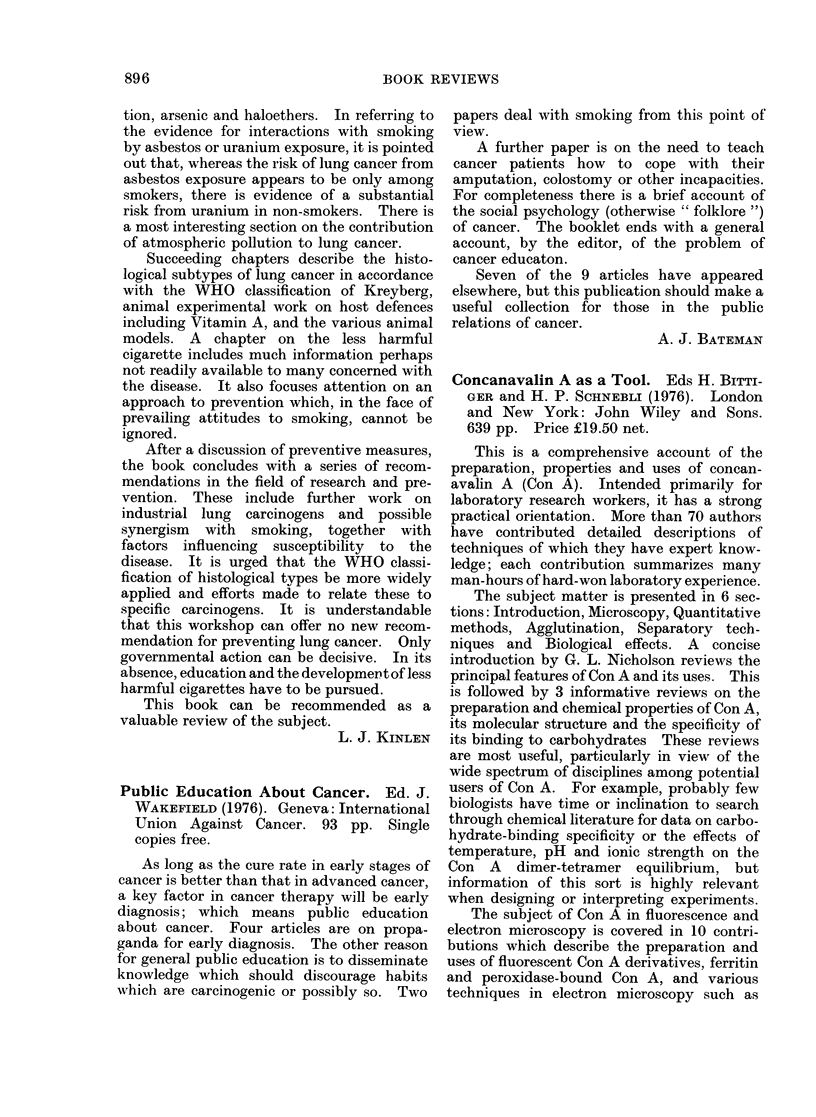# Public Education About Cancer

**Published:** 1977-06

**Authors:** A. J. Bateman


**Public Education About Cancer.** Ed. J. WAKEFIELD (1976). Geneva: International Union Against Cancer. 93 pp. Single copies free.

As long as the cure rate in early stages of cancer is better than that in advanced cancer, a key factor in cancer therapy will be early diagnosis; which means public education about cancer. Four articles are on propaganda for early diagnosis. The other reason for general public education is to disseminate knowledge which should discourage habits which are carcinogenic or possibly so. Two papers deal with smoking from this point of view.

A further paper is on the need to teach cancer patients how to cope with their amputation, colostomy or other incapacities. For completeness there is a brief account of the social psychology (otherwise " folklore ") of cancer. The booklet ends with a general account, by the editor, of the problem of cancer educaton.

Seven of the 9 articles have appeared elsewhere, but this publication should make a useful collection for those in the public relations of cancer.

A. J. BATEMAN